# Beyond recidivism: reconceptualizing success through relational health for trauma-exposed youth experiencing juvenile justice involvement

**DOI:** 10.3389/fpsyg.2024.1263451

**Published:** 2024-02-27

**Authors:** Amanda D. Zelechoski, Janet Bohner, Bruce D. Perry

**Affiliations:** ^1^Department of Psychology, Purdue University Northwest, Hammond, IN, United States; ^2^Indiana Department of Child Services, Indianapolis, IN, United States; ^3^School of Allied Health, Human Services and Sport, La Trobe University, Melbourne, VIC, Australia; ^4^Department of Psychiatry and Behavioral Sciences, Feinberg School of Medicine, Northwestern University, Chicago, IL, United States

**Keywords:** trauma, juvenile, trauma-informed, recidivism, relational health

## Abstract

Youth who have histories of trauma exposure face unique barriers and needs in navigating the juvenile justice system. Accordingly, reliance on recidivism as the primary “success” metric falls short for trauma-impacted youth and may actually prolong their justice involvement. Caregivers and juvenile justice professionals (i.e., judges, attorneys, detention and probation staff, case managers, and mental health clinicians) often struggle to identify and adequately address these challenges and pitfalls. This policy brief provides an overview of specialized considerations for traumatized youth with respect to common policies and practices, namely mandated placement, treatment, and timelines. Specific examples and actionable recommendations are provided to assist juvenile justice professionals and treatment providers with systemic efforts to more appropriately and effectively customize juvenile justice policies and programs for these extremely vulnerable youth.

## Introduction

*We have few metrics to measure the number, quality, and patterns of healthy (or unhealthy) relational interactions; we move traumatized children from therapist to therapist, school to school, foster home to foster home, community to community. Indeed our systems often exacerbate or even replicate the relational impermanence and trauma of the child’s life*. (Ludy-Dobson and Perry, 2010, p. 39)

**Figure 1:**
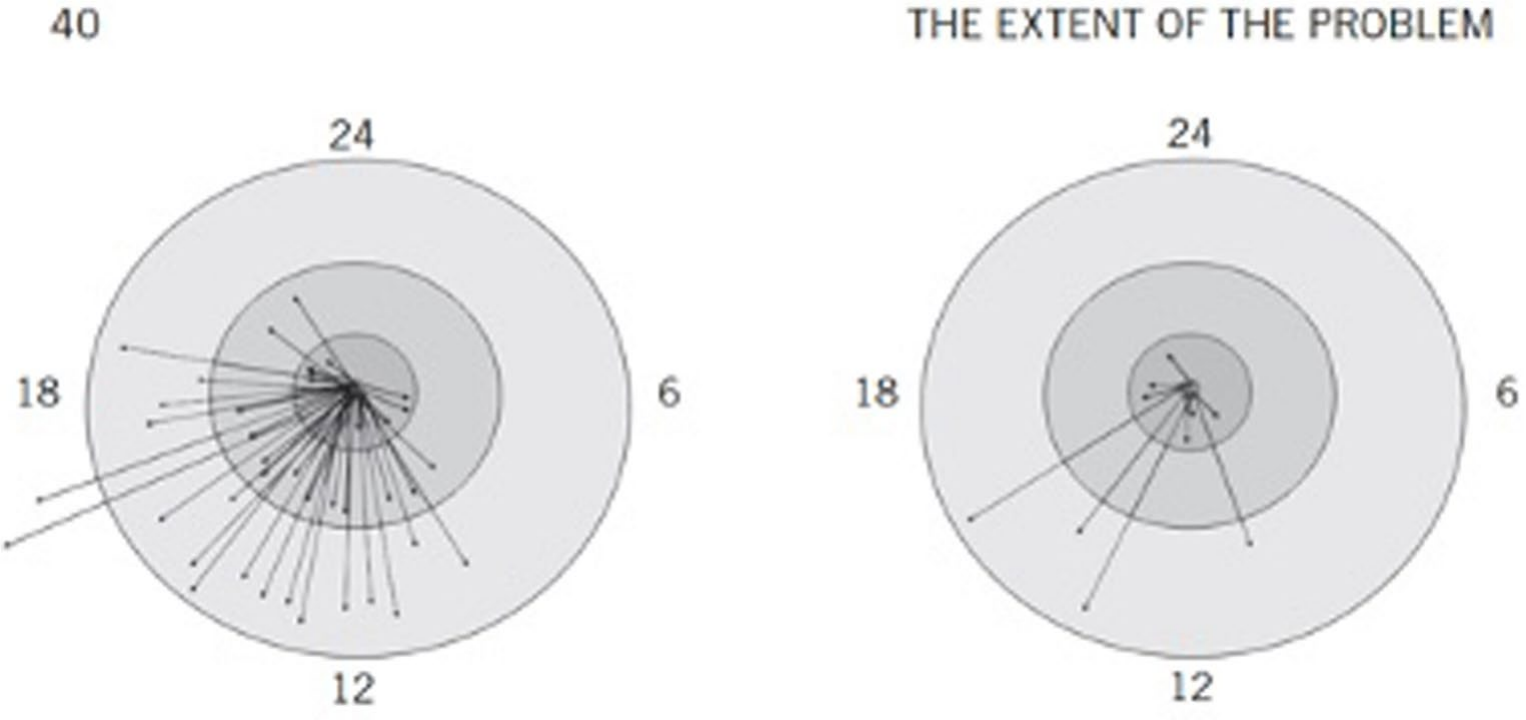
Case comparison of positive relational interactions used with permission of Guilford Publications, from Ludy-Dobson and Perry (2010) permission conveyed through Copyright Clearance Center, Inc. Positive relational interactions: Typical and foster child. These two figures are representative 24-hour relational contact maps examining the number of positive relational interactions in two children. Arrows represent positive interactions (as rated by observer and child); arrows ending in the inner circle represent interactions with family; additional circles represent friends, then classmates/acquaintances. Arrows outside the circle represent interactions with strangers. The figure on the right is based on a 10-year-old boy in foster care who was moved in the middle of the school year to a new foster home away from extended family and community. This figure is the best 24-hour map for a 2-week period for this child. Several days were completely devoid of any positive relational interaction. The relational poverty played a major role in this child’s inability to progress; symptoms related to trauma and neglect persisted and increased while he was in relationally impoverished settings. Once in a stable placement with positive relationships created in school and the community, he stabilized and improved.

In 2010, Ludy-Dobson and Perry published data from a study in which they captured the nature and quantity of positive relational interactions between a “typical” child (i.e., without significant mental health issues and living with biological parents) and a child in foster care over a 24-h period (see Figure 1). The contrast between the two children’s daily experiences was striking. The “typical” child was growing up in a relationally enriched environment, full of “dots” or frequent, positive interactions with family, peers, acquaintances, and community members. The foster child’s best 24-h relational contact map in the two-week observational period had very few dots – some days had zero positive relational interactions, zero dots. Ludy-Dobson and Perry explained that the relational poverty this foster child experienced was likely the primary reason for his inability to stabilize and improve. He had lots of interactions with others throughout his day, but very few were positive, which mirrors the chronic daily experiences of many trauma-exposed, juvenile justice-involved youth.

It is well-established that youth in the juvenile justice system are among the most traumatized and vulnerable populations (Abram et al., 2013; Zelechoski, 2016). Research has demonstrated an increasingly robust empirical relationship between type of childhood trauma exposure and subsequent delinquent behavior types and patterns (Dierkhising et al., 2013). Developmental or complex trauma is particularly prevalent among justice-involved youth, leading to numerous interpersonal and emotion regulation difficulties (Ford et al., 2012). Consequently, failure to implement developmentally-aware and trauma-sensitive policies and practices may not only be ineffective juvenile justice policy, but also exacerbate prior adversity and cause additional harm for justice-involved youth (Perry et al., 2018).

One such potentially outdated policy may be the steadfast focus on juvenile recidivism as the primary metric for youths’ success, regardless of whether the local or national pendulum is currently leaning toward punitive versus rehabilitative practices. Recidivism or “reversion of an individual to criminal behavior” (Maltz, 1984, p. 1) has long been the standard outcome measure in juvenile justice research and policy. However, despite its popularity, there remain numerous inconsistencies in operational definitions and measurement, as well as criticisms of using recidivism as the sole measure of effectiveness for juvenile justice decision-making, placement, and treatment (e.g., Steinberg, 2017; Caudill and Trulson, 2022). In fact, well-known mental health advocate, Sharon Wise (2014), stated, “You call it recidivism, I call it continuum of care; someone keeps coming back to you because they need something from you. It’s not recidivism, it’s not relapse. It’s going back to continue the care.”

Accordingly, what if we turned our usual model on its head – shifted our focus away from decreasing recidivism and instead toward increasing relational health, or the quality of relationships across multiple contexts, for justice-involved youth? What if we focused less of our efforts and resources on what we do not want youth to do and invested more in surrounding youth with stable, consistent, and positive relationships? Such an approach would be consistent with research demonstrating that positive relationships can be protective against traumatic stress (e.g., Pat-Horenczyk et al., 2008), as well as the recent call by the American Academy of Pediatrics for “a paradigm shift toward relational health because safe, stable, and nurturing relationships not only buffer childhood adversity when it occurs but also promote the capacities needed to be resilient in the future,” (Hambrick et al., 2021, p. 1, quoting Garner et al., 2021, p. 1). However, as Hambrick et al. (2021) cautioned, “we cannot just say ‘provide children with better relationships’ without developing systems that prioritize relationships…” (p. 2). Accordingly, we briefly describe juvenile justice policy considerations and implications, as well as actionable recommendations for juvenile justice and mental health professionals to more effectively and meaningfully measure success for trauma-exposed youth.

## Policy considerations and implications

### Mandated placements

In 2019, 36,479 youth were placed in out-of-home or residential placement as part of their juvenile justice involvement, either during their pre-adjudication detention period or as their post-adjudication disposition placement. Of those youth, 41% were Black, 85% were male, and more than half were aged 16 or 17 (Hockenberry, 2022). This 2019 Office of Juvenile Justice and Delinquency Prevention (OJJDP) census total represents a 65% decrease in residential placement for justice-involved youth since 1997; however, considering the majority of these youth spend more than 4 months, on average, in residential placement, the potentially negative long-term developmental, educational, and psychological impacts are profound (Zelechoski et al., 2013). Hockenberry also noted that, though 93% of youth were placed in facilities in the same state in which they committed their offense (presumably their home state), states vary considerably in terms of number of residential facilities and beds; thus, many of these youth were likely placed a substantial distance from their homes and communities. This high proportion of youth being held in out-of-home, long-term, restrictive settings is concerning for several reasons.

First, the way mandated placement decisions are made by judges or recommended by probation officers are often inconsistent and problematic. In fact, McPhee et al. (2023) found that “life-altering decisions regarding confinement appear to be made—at least in part—on the basis of individual decisionmakers’ idiosyncratic interpretations of a youth’s potential risk of committing another crime in the following year, impressions that do not appear to be grounded in accurate data or to reflect realistic appraisals” (p. 328). Second, the contingency management structures of many residential treatment programs are likely counterproductive for many youth with histories of traumatic exposure (Perry and Ablon, 2019). Specifically, the point and level systems utilized by many residential treatment facilities are often experienced by youth as rigid and punitive, particularly for those struggling with low frustration tolerance and minimal emotion regulation capacities, essentially setting up dysregulated youth to fail (see, e.g., Mohr et al., 2009; Perry et al., 2018). Third, the abrupt and intensive participation in individual therapy required in most residential placements can be extremely triggering for youth who have experienced complex or developmental trauma and, thus, struggle with attachment, trust, and vulnerability with others. In fact, research has demonstrated that individual therapy can be effective for youth with significant relational poverty (few meaningful connections to other people), but may have minimal or even negative impact for youth who already have a consistent, supportive, and relationally-enriched community (Renick, 2018; Johnson-Kwochka et al., 2022). Finally, high levels of turnover, absenteeism, and burnout among residential placement staff are directly related to increased juvenile risk, recidivism, and treatment failure (Wolff et al., 2022), likely because such staffing difficulties result in abrupt transfers of care and a chaotic and unpredictable “therapeutic web” or “social milieu”– the opposite of what trauma-exposed youth need (Ludy-Dobson and Perry, 2010).

### Mandated treatments

It is well established in U.S. law and policy that the juvenile justice system is intended to be more rehabilitative than punitive. Whether and how that plays out in practice and reality varies widely across jurisdictions, particularly for youth with concurrent developmental delays and/or mental health issues. Due process requires that the nature of any mandated treatment be reasonably related to the purpose for which the individual is confined (e.g., Jackson v. Indiana, 1972), and yet there is still discrepancy across jurisdictions with respect to whether they explicitly acknowledge a right to mental health treatment for justice-involved youth and the appropriateness of specific treatments they mandate relative to nature of youth’s delinquent behavior (Mulford et al., 2004). The evidence-based treatment modalities to which many justice-involved youth are mandated often involve engaging in top-down cognitive processes and/or discussing intimate aspects of their early adverse experiences with a stranger (albeit a well-meaning mental health or juvenile justice professional) before youth are emotionally and physiologically regulated enough to do either (Perry et al., 2018). Thus, we again set trauma-exposed youth up to fail and classify them as “treatment-resistant,” when it is more likely that the nature, timing, and duration of the therapeutic interactions required by many evidence-based interventions will be experienced as triggering, threatening, and dysregulating and result in self-protective and, often, aggressive behaviors (Berko, 2021).

This is consistent with Perry et al.’ (2018) emphasis on understanding relational neurobiology before making specific juvenile justice treatment decisions:

For individuals with relational histories of inconsistent or abusive care (all too common in youth and adults in the justice system), relational associations will be negative; interacting with others will likely be threat inducing and dysregulating. Intimacy becomes associated with threat and loss, not comfort and safety. This has profound implications; among them is an alteration of the sense of personal space…[and] an inter-related concept, the intimacy barrier, [which] focuses on both personal and emotional space boundaries. When the intimacy barrier is crossed without permission (e.g., …for a child in the child welfare system someone asks about your family), the individual feels threatened. The stress response systems (including the amygdala) activate and the individual will engage in protective behaviors…it can be very confusing for peers, carers, and educators when their intended nurturing behaviors and words are met with overt hostile and aggressive behavior or indifferent and dismissive attitudes. (p. 825–826).

When mandating justice-involved youth into treatment, perhaps the focus then should be less on the specific treatment method or program and more on with whom a trauma-exposed youth is ordered into treatment, as well as identifying concrete ways to gradually increase that youth’s “therapeutic web” or network of strong, consistent, and meaningful relationships. In their study of over 100 juveniles court-ordered into treatment, Farrouki and Mapson (2007) found that the effectiveness of court-mandated treatment in reducing recividism depended, in large part, on the strength and quality of the relationship between the justice-involved youth and the clinician, not on the specific treatment modality. The importance of giving trauma-exposed individuals some control over the timing, dosing, spacing, and individuals with whom they are willing to cross their intimacy barriers is well-established (Perry et al., 2018; Berko, 2021). Accordingly, mandated treatment decisions should prioritize relationships with developmentally-informed, trauma-competent professionals who can effectively manage the complexity of intersectional factors for youth (e.g., age, gender, race), over specific intervention models. “People, not programs change people,” (Perry and Szalavitz, 2017, p. 85).

### Mandated timelines

The third policy area for consideration is legally-imposed or mandated intervention timelines, particularly for trauma-exposed youth, as there are practical concerns on both ends of the spectrum. For example, the second author recently made the following statement during court testimony on behalf of a justice-involved youth, “[She’s] only been in therapy once a week for four months; [you] want miracle-type results when this girl has had years and years of trauma built up.” Similarly, the third author expressed frustration about unreasonable timelines and expectations in a media interview, “You cannot have a kid who’s lived with daily humiliation and neglect for six years and think your authorization for 20 sessions [of therapy] is going to fix the problem. It’s not, and it’s bullshit,” (Renick, 2018). Though systemic efforts to expedite the process and divert youth out of the juvenile justice system are commendable, they can result in inappropriate dosage and intensity of the required intervention, which can, again, set youth up for failure and further exacerbate their shame, stigma, and mistrust of mental health treatment and clinicians.

On the other extreme, over 1/3 of justice-involved youth spend more than six consecutive months in court-ordered out-of-home placements (Hockenberry, 2022). This may seem like a relatively brief period but, when using a developmentally-informed perspective, this is an extremely long stretch of time to disrupt attachments with primary caregivers and positive relational connections in the community to which the youth will return and be expected to immediately reintegrate and thrive. Accordingly, it is critical to understand that one size does not fit all, though juvenile probation guidelines, policies, and practices in some jurisdictions would potentially imply otherwise. Projected timelines and expectations need to be individualized, flexible, developmentally matched, and appropriately dosed for trauma-exposed youth (Perry et al., 2018). We provide some specific recommendations and considerations for doing so below. These recommendations are intended for individual juvenile justice professionals (i.e., judges, attorneys, detention and probation staff, case managers, and mental health clinicians), as well as systemic and cross-system consideration (e.g., juvenile justice, child welfare, education).

## Actionable recommendations

1. *Create a plan that sets youth up to succeed and increases their “dots.”*

It is important to consider a youth’s current relational environment before mandating intervention placements, treatment types, or timelines. “We expect ‘therapy’—healing—to take place in the child via episodic, shallow relational interactions with highly educated but poorly nurturing strangers. We undervalue the powerful therapeutic impact of a caring teacher, coach, neighbor, grandparent, and a host of other potential ‘co-therapists,’ (Ludy-Dobson and Perry, 2010, p. 39). Research has demonstrated the benefits of group and family intervention modalities over individual therapy structures for justice-involved youth with relationally-enriched environments (Johnson-Kwochka et al., 2022). Accordingly, if the trauma-exposed youth has a strong and consistent therapeutic web, consider how you can leverage and strengthen those existing relationships within the probation or intervention plan, rather than creating a structure that disrupts meaningful and safe connections in the youth’s home and community.

2. *Re-evaluate “amenability to treatment” with a trauma-informed lens.*

When justice-involved youth refuse to meet with clinicians, storm out of therapy sessions, disrupt group therapy dynamics, or demonstrate any number of additional behaviors that do not immediately signal compliance and improvement, we tend to classify them fairly quickly and systemically as “not amenable to treatment” or “non-compliant.” This classification can have significant implications in terms of legal disposition and placement, as well as create bias and more barriers for establishing therapeutic rapport and trust in the future. In addition to the previously discussed intimacy barriers, many justice-involved youth have understandable and justifiable reasons for mistrusting juvenile justice and mental health professionals. These include viewing clinicians as part of the legal system that has separated them from their families, peers, and communities, protecting oneself from people or systems that the youth perceives as oppressive to the youth’s family or culture, or complying with prior familial or cultural conditioning and stigma that talking to a clinician is shameful or violates others’ privacy. Youth with marginalized identities (i.e., youth of color, disabled youth, LGBTQ youth) are often characterized as non-compliant and/or not amenable to treatment in situations in which the treatment was neither trauma-informed nor culturally-competent (Venable and Guada, 2014). For both juvenile justice decisionmakers and treatment providers, rather than presuming that something is wrong with the justice-involved youth when therapeutic gains are not being made, consider whether something might instead be misaligned in terms of the therapeutic relationship, specific intervention methods being utilized, or lack of cultural responsivity.

3. *Reconsider “disruptive” and “maladaptive” behaviors.*

For many trauma-exposed youth, what are often considered maladaptive behaviors have actually served important adaptive functions in situations where youth had no alternative coping strategies upon which to rely for their survival. Further, the rules and restrictions inherent in many probation and placement settings may take away positive coping strategies youth have previously used. For example, being able to walk away from a volatile interaction with an adult to emotionally and physiologically regulate oneself may be perceived as disrespectful and result in punishment or dropping down a level in contingency management programs. Similarly, leaving the house to de-escalate and get some space from a triggering family member may be prohibited by probation restrictions. Further, if youth return home from placements having successfully employed positive coping strategies and caregivers do not have the capacity to or are not given the proper tools to maintain those strategies in the home, the youth will likely escalate and regress to prior “maladaptive” behaviors, because they are familiar and have been adaptive in the past. As Berko (2021) noted, for many traumatized youth, “…the lack of progress on a behavioral incentive plan reflects more than just resistance” (p. 10). To put it simply, get curious, not furious, about youths’ seemingly maladaptive behaviors. They likely serve a function for that youth in that situation. The more you can try to authentically explore and understand what that function is, the more you can collaborate with the youth to identify feasible and sustainable alternative strategies.

4. *Allow for individualized, flexible, dosed, and modifiable probation orders and treatment plans.*

The child welfare and juvenile justice systems are often tasked with developing and relying on population-level solutions, rather than employing individualized approaches. Similarly, the U.S. legal system focuses primarily on punishing the offense committed, rather than on addressing the underlying reasons for the behavior that led to the offense. We hope the empirical context we have provided thus far has made it clear that what trauma-exposed, justice-involved youth need are more individually-tailored approaches to their rehabilitation. This includes probation and treatment plans developed by or in partnership with trauma-competent clinicians that understand the degrees of flexibility and relational dosing needed, as well as the importance of incorporating opportunities for trauma-exposed youth to exercise some control over their intimacy barriers. For example, rather than a probation plan specifying that a youth must participate in “2 h of individual therapy per week” (which is often interpreted to mean two 60-min sessions of top-down/cognitively-loaded verbal interaction), the plan specifies that therapist and youth will agree on a graduated plan for increasing the number of minutes per week spent working together and the youth has some autonomy in when and how they “spend” those minutes during times when they are feeling regulated and able to engage with the therapist (see, e.g., Berko, 2021 and Barrett and Rappaport, 2011 for additional clinical examples of flexibility with trauma-exposed, justice-involved youth).

Trauma-informed juvenile justice professionals understand that relational interactions are often triggering for youth, particularly given that the vast majority of justice-involved youths’ traumatic experiences took place in the context of their intimate relationships, and they are now being forced into another intimate relationship with a stranger (therapist). Because of their histories of trauma, their brains are “wired” to experience this as dangerous and, thus, to avoid or fight it. However, trauma-exposed youth also deeply fear abandonment. So, if they know that their relationships with therapists or juvenile justice staff members are time-limited or (because of previously discussed high turnover rates) will likely be abruptly cut off, they are understandably hesitant to invest in the relationship and allow themselves to be vulnerable.

Flexible and individualized probation orders and treatment plans also recognize that timelines will vary drastically across youth. As Berko (2021) illustrated through several powerful case examples, many justice-involved youth will regress and show signs of getting worse before they start to get better because they are “actively repairing developmental deficits” (p. 10). Thus, we need to be patient and allow for the cycles of ruptures and repairs that are needed for youth to start to gradually establish a sense of safety in the vulnerability of therapeutic work. This does not necessarily mean that youth need to be in out-of-home placements for longer durations; rather, it simply means that timelines should (1) remain flexible; (2) be guided by the trauma-exposed youth’s needs and capacity of the involved systems and communities to meet those needs; and (3) prioritize minimal separation of youth from relationally healthy environments.

## Conclusion

In this policy brief, we sought to expand the juvenile justice system outcome metric beyond recidivism to incorporate more holistic conceptions of relational health and youth well-being as measures of success. Shifting focus and resources toward increasing the nature, quantity, and frequency of positive relational interaction “dots” for justice-involved youth is consistent with current pediatric, education, mental health, and public health policy efforts. In addition to reducing delinquent behavior and out-of-home placements (and thus reducing costs), policies and practices that are trauma responsive and intentionally address relational poverty will go a long way toward promoting long-term healing and empowerment for our most vulnerable youth.

### Additional resources

The following is a list of additional resources relevant to this study:

– Cruise et al. (2019).

– Griffin, G et al. (2012).

– National Child Traumatic Stress Network, Justice System Consortium (2009).

– National Child Traumatic Stress Network and National Council of Juvenile & Family Court Judges (2013).

– National Child Traumatic Stress Network (2015).

– National Child Traumatic Stress Network, Justice Consortium Attorney Workgroup Subcommittee (2018).

– National Child Traumatic Stress Network (2021).

## Author contributions

AZ: Conceptualization, Writing – original draft, Writing – review & editing. JB: Conceptualization, Writing – review & editing. BP: Conceptualization, Writing – review & editing.
